# Comparison of intravenous sedation using midazolam versus dexmedetomidine in elderly patients with dementia: a randomized cross-over trial

**DOI:** 10.1038/s41598-022-10167-2

**Published:** 2022-04-15

**Authors:** Yoshinari Morimoto, Megumi Hayashi, Yuki Yao, Hitomi Nishizaki, Hidechika Ishii, Lou Mikuzuki, Kouji Hara

**Affiliations:** Division of Geriatric Dentistry, Department of Critical Care Dentistry, Kanagawa Dental University, 82, Inaoka-cho, Yokosuka, Kanagawa 238-8580 Japan

**Keywords:** Diseases, Medical research, Neurology

## Abstract

Differences between the effects of intravenous sedation with midazolam (MID) and dexmedetomidine (DEX) on the cerebral function of elderly patients with severe dementia are unclear. This study aimed to compare the effects of intravenous sedation with MID or DEX on parameters such as brain waves and cerebral blood flow (CBF). This cross-over study analyzed 12 patients with severe dementia, with each patient receiving both drug treatments. Each drug was administered until a Modified Observer’s Assessment of Alertness/Sedation (OAA/S) score of 2 was reached. Bispectral index (BIS) and normalized tissue hemoglobin index (nTHI), which reflects CBF using near-infrared spectroscopy, were measured. Mann–Whitney U, Wilcoxon signed-rank, and Friedman tests, and multiple regression analysis were performed. While a similar decline in BIS values was observed in both groups (P < 0.030), there was a significant decrease in nTHI up to 11% in the MID group (P = 0.005). In the DEX group, nTHI values did not differ from baseline. When an OAA/S score of 2 was just achieved, CBF in the MID group (− 5%) was significantly lower than in the DEX group (± 0%). In dementia patients, sedation with MID resulted in a decrease in CBF, while the CBF value was maintained during sedation with DEX.

## Introduction

Intravenous sedation is used as a behavior modification technique during the dental treatment of patients with severe dementia who offer resistance or refuse to undergo dental treatment. Midazolam (MID), dexmedetomidine (DEX) and propofol are often used for intravenous sedation during dental treatment^1^. MID is classified as a benzodiazepine sedative and, like the other benzodiazepines, is a gamma-aminobutyric acid (GABA) receptor agonist that is thought to induce cognitive impairment, such as postoperative delirium (POD)^2–4^. Propofol, another GABA receptor agonist, is thought to exert similar effects^2–4^. On the other hand, unlike GABA receptor agonists, such as propofol and benzodiazepine sedatives, DEX acts on central α2-adrenergic receptors. Sedation with DEX has a minimal effect on respiratory rate and percutaneous arterial oxygen saturation (SpO_2_), and is known for the associated fast recovery from sedation with physical stimulation if needed^5–9^. It might be an appropriate sedative for elderly patients with dementia since it is less likely to inhibit the gag reflex, and animal studies have shown that it has cerebral protective effects, such as maintenance of cerebral blood flow (CBF) during hypoxia and focal cerebral ischemia, and causes reduction of brain cell damage^5–9^.

Cognitive impairments that develop after surgery are classified as POD and postoperative cognitive dysfunction (POCD). Many studies have found that preoperative cognitive decline, in particular, has an effect on the onset of POD and POCD^2–4^. One of the factors that might trigger POD and POCD is surgery-induced inflammation that spreads to the central nervous system^2–4^. DEX has an anti-inflammatory effect, and studies have shown that it has a low incidence rate of POD and POCD after cardiac and non-cardiac surgeries^5–9^.

It is generally believed that maintaining neuronal activity (brain waves and CBF) under anesthesia is important for maintaining perioperative cognitive function^2–4^. During intravenous sedation with midazolam in elderly patients with severe dementia, dose titration to a lower than the usual dose is needed, and respiratory complications (apnea) have occasionally been reported in this population. These conditions are different from those with other sedatives, such as propofol and dexmedetomidine^10^. In our previous prospective study, we found that CBF in elderly patients with no cognitive decline remained stable after intravenous sedation with 0.035 mg/kg of MID. However, when 0.027 mg/kg of MID was administered to elderly patients with severe dementia, CBF decreased by approximately 10%^11^. As the majority of dental treatments are conducted over the course of multiple visits, notable cognitive decline is possible from repetitive use of intravenous sedation. Hence, we need to identify sedatives that do not affect cerebral function, and DEX seems to potentially be one such drug.

This study prospectively registered elderly patients with severe dementia who were scheduled to undergo behavior modification with intravenous sedation for dental treatment. The objective of the present study was to investigate and compare the effects of intravenous sedation with two types of drugs, MID and DEX, on brain waves, assessed as the bispectral index (BIS) value, and CBF in each subject.

## Methods

### Study design

This study was conducted in accordance with the Declaration of Helsinki. The study procedures were approved by the Institutional Research Board (IRB) and Ethics Committee of Kanagawa Dental University (Approval No. 472). The procedures, benefits, safety and risks of the present study were thoroughly explained to each of the subjects and/or their guardians, and their written informed consent was obtained. The research plan for this study was registered and published in the University Hospital Medical Information Network Center (UMIN Clinical Trails Registry) (ID: UMIN000036530; registered on 16, April, 2019).

### Study participants

The subjects were patients with severe dementia aged 60 years and over who visited the geriatric dental clinic at Kanagawa Dental University Hospital between April 2019 and March 2021, and were classified as physical status (PS) class 1 or 2 of the American Society of Anesthesiologists (ASA) classification, except for the presence of severe dementia. Of these patients, those who were scheduled to undergo two or more dental treatments (including dental extraction) using intravenous sedation with MID or DEX due to being uncooperative for dental treatments because of advanced dementia were selected as subjects. Once the schedule of sedation was established, all the subjects were assessed using the Mini-Mental State Examination (MMSE). Subjects with an MMSE score of 23 or less, or those who could not undergo the MMSE test due to severe cognitive decline were considered to have dementia^12^. Among elderly patients with dementia, those who met the following criteria for “severe dementia” were registered in the present study: (i) patients with Functional Assessment Staging of Alzheimer’s Disease (FAST) stage 6 or 7 among the Alzheimer-type dementia patients and/or (ii) patients with a Clinical Dementia Rating (CDR) of “severe” in all categories among all patients, including those with other types of dementia. MMSE, FAST and CDR were assessed by a separate medical specialist (LM) and the results of testing were not disclosed to the attending anesthesiologists (YM and MH). The exclusion criteria for subjects were (i) patients without cognitive impairment (MMSE ≥ 24); (ii) patients whose dementia was not severe (MMSE ≤ 23 but with a FAST score of ≤ 5 and/or CDR ≤ 2), (iii) patients with allergies, glaucoma, etc. who were contraindicated for MID or DEX, (iv) patients with anemia, with Hb < 10 g/dL, and (v) patients with diseases other than dementia, equivalent to ASA PS 3 or higher. Two of the participants previously had episodes of hypertension, but were not on any antihypertensive medication since their blood pressure had decreased to age-appropriate levels; these patients were included in the present study. There were no participants with diabetes mellitus.

**Figure 1:**
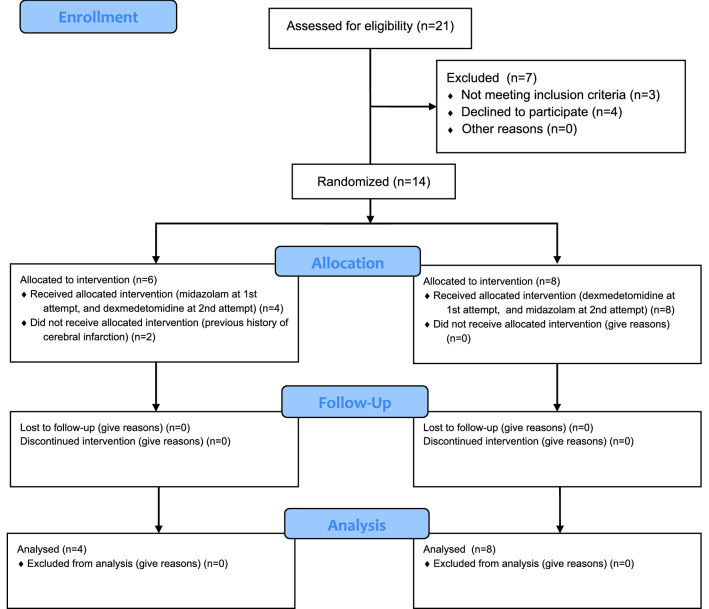
CONSORT flow diagram showing the selection process of patients.

The study procedures were explained to all the patients and/or the guardians of patients who met the above-mentioned inclusion criteria during the study period. A total of 14 patients who gave consent for participation were registered. The present study adopted a cross-over study design, where the same patient received one of the two drug treatments in random order during separate treatment sessions. Elderly patients with advanced dementia were randomized into two groups based on the order of administering MID and DEX determined using a computer-generated random number: the MID/DEX group (patients received MID for the first procedure and DEX for the second procedure); and the DEX/MID group (patients received DEX for the first procedure and MID for the second procedure). Six patients were randomly registered in the MID/DEX group and eight patients in the DEX/MID group based on the randomization process (Fig. 1). However, after the allocation, two patients in the MID/DEX group were found to have had a history of cerebral infarction without paralysis sometime in the previous few years. Since the data of cerebral function (cerebral blood flow and brain waves) can be inaccurate in such patients, these two participants were excluded from data collection (Fig. 1).

Intravenously administered MID and DEX are eliminated from the body after 24 h and 72 h, respectively^13,14^. Based on pharmacodynamic principles, a wash-out period of three or more weeks was established between the administration of each drug. Group assignments were managed independently by a researcher (LM) other than the anesthesiologists. All the measurements and records from the near-infrared spectroscopy (NIRS) probes, BIS values and OAA/S scores under sedation were concealed from the attending anesthesiologists as they were measured and recorded by researchers other than the attending anesthesiologists (YY and HN).

### Recordings

#### Monitoring and sedation methods

Based on the “Practice Guidelines for Intravenous Conscious Sedation in Dentistry” published by the Japanese Dental Society of Anesthesiology, the study participants were asked to refrain from eating for 8 h and from drinking two hours prior to the start of sedation^1^.

Preanesthetic medications were not used. After entering the treatment room, the patients were asked to sit on the treatment table and were then placed in the horizontal position. A non-invasive blood pressure monitor, electrocardiograph and SpO_2_ monitor, as well as BIS sensors and NIRS probes [NIRO^®^-200NX (Hamamatsu Photonics Co., Hamamatsu, Japan)], were placed on the forehead^15^. A nasal cannula for carbon dioxide (CO_2_) monitoring (Smart CapnoLine^®^ plus O_2_; Covidien Japan, Tokyo, Japan) was applied and end-tidal carbon dioxide partial pressure (etCO_2_) was measured using Capnostream^TM^20P (Medtronic Covidien Japan Co., Tokyo, Japan). The nasal cannula of this device that is used to measure etCO_2_ also allows simultaneous oxygen delivery. As the data measurements in the present study were taken before starting the dental treatment, the assistant lightly lifted up the chin of the patient to manually guide light closure of the patient’s mouth, to ensure that CO_2_ measurements were taken with the patient breathing only through the nose.

**Figure 2:**
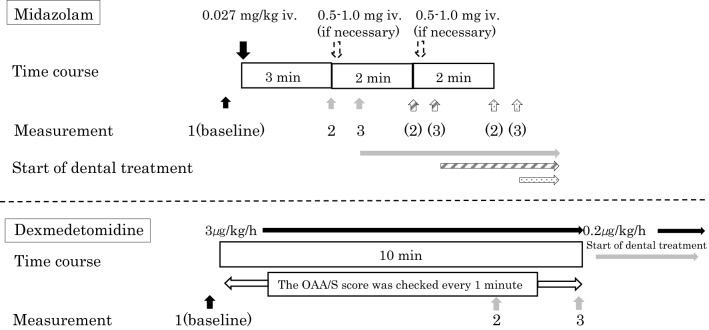
Time course of midazolam or dexmedetomidine administration and measurement of physiological parameters. In the midazolam group, a Modified Observer’s Assessment of Alertness/Sedation (OAA/S) score of 2 was maintained at both the measurement points 2 and 3. In the dexmedetomidine group, an OAA/S score of 2 was achieved at measurement point 2. However, OAA/S scores of both 2 and 1 were observed at measurement point 3 due to deepening of the sedation level in four patients.

A peripheral intravenous line was secured using a 22G intravenous cannula at the dorsum of the right or left hand. The administration of 2 mL/kg/h of acetated Ringer’s solution (Fisio 140^®^: Otsuka Co. Tokyo, Japan; 1% glucose and acetated Ringer’s solution) was started using an infusion pump. Midazolam has a rapid onset of action and its peak effect is reached within 2–3 min following administration. After intravenous administration, midazolam is rapidly distributed, with a distribution half-life of 6–15 min^16^. Based on these pharmacological data, in our previous study we found that intravenous sedation with 0.027 mg/kg (median value) MID administration in elderly patients with severe dementia resulted in achievement of a Modified Observer’s Assessment of Alertness/Sedation (OAA/S) score of 2 (responds only after mild prodding or shaking)^11^. Therefore, in the present study, the patients were given 0.027 mg/kg of MID and were observed for 3 min to ensure that an OAA/S score of 2 was achieved. If an OAA/S score of 2 was not achieved by this point, 0.5 to 1 mg of MID was administered intermittently until an OAA/S score of 2 was reached (Fig. 2)^11^. On the other hand, DEX (6 μg/kg/h given for 10 min) has a slower onset of intraoperative sedation than propofol^16^. The average intraoperative infusion rate of DEX required to maintain a BIS value of 70 to 80 is reportedly 0.7 μg/kg/h, and its elimination half-time is 2–3 h^16^. Based on these pharmacological facts, the drug manual recommends administering 6 μg/kg/h DEX (or 3 μg/kg/h for elderly patients) for 10 min as the initial dose, followed by 0.2–0.7 μg/kg/h for maintenance^14^. In accordance with the package insert, DEX was started at an initial dose of 3 μg/kg/h for 10 min, followed by a dose of 0.2 μg/kg/h, at which point dental treatment was commenced (Fig. 2). In our previous report on sedation for patients with severe dementia, an SpO_2_ of < 94% was observed in approximately one-third of the cases when DEX was used, with most of them occurring during the latter 5 min of administration of the initial load (total duration: 10 min) of 3.0 mg/kg/h, at which time their BIS value was approximately 33–45. These patients could not respond to mild prodding and shaking, which was equivalent to an OAA/S score of 1^10^. DEX is generally known to be associated with fast recovery from sedation with physical stimulation, if needed^16^. In our severe dementia patients, because OAA/S scores of 2 or 1 were reached during initial dose administration, the patients’ sedation levels were checked every 1 min for 10 min, and slow or subtle responses to mild prodding and shaking were defined as an OAA/S score of 2. The evaluation of an OAA/S score was continued until treatment initiation.

Dental treatment was commenced when the DEX dose was changed to the maintenance dose of 0.2 μg/kg/h (Fig. 2). For evaluation of the OAA/S score, the researchers loudly and repeatedly called out the patient’s name (or other words that the patients could respond to, as reported by their guardians to the researchers beforehand), followed by prodding or shaking, and their responses were observed to determine whether their sedation level reached an OAA/S score of 2. The duration of calling and/or prodding was performed within 10 s per min, which was considered not to impede the deepening of sedation level.

After sedation, the patients were allowed to breathe room air unless SpO_2_ decreased to 90% or less. If SpO_2_ went below 90%, oxygen was delivered at 1 L/min via the nasal cannula. When an SpO_2_ of < 90% persisted, an additional 1 L/min of oxygen was added until an SpO_2_ of ≥ 90% was reached.

When measurements were taken with NIRO^®^-200NX, the following factors that affect cerebral tissue normalized tissue hemoglobin index (nTHI) and cerebral tissue oxygenation index (TOI) measurements were avoided: (i) anemia, with Hb < 10 g/dL; and (ii) probe placement on forehead veins. Additionally, measurements were made after the subjects rested for 5 min in the horizontal position^15^.

#### Measurement parameters and measurement period

Information on the patients’ age, sex, height, weight and body mass index (BMI) were collected as patient background data from their medical records. Duration of treatment and sedation time were investigated as parameters related to the sedation method. The physiological parameters measured under sedation were mean arterial pressure (MAP), SpO_2_, etCO_2_, BIS value and NIRO^®^-200NX measurements [right and left cerebral TOI and nTHI]^15^.

During sedation, taking into consideration that, for MID, a clinical response is achieved when blood concentrations are highest, approximately after 3 min of MID administration, TOI and nTHI in the MID group were measured at the following three measurement points: (i) 5 min after commencing measurements (the time period required for stabilization of measurements) and just before the start of MID administration (TOI 1, nTHI 1); (ii) when an OAA/S score of 2 was reached after MID administration (TOI 2, nTHI 2) and (iii) just before the start of treatment (TOI 3, nTHI 3)^16^. In the DEX group, while maintaining the infusion rate of DEX in the range of 0.2 to 0.7 μg/kg/h after the initial loading dose of 3 μg/kg/h for 10 min, measurements were taken at the following three measurement points: (i) 5 min after commencing measurement (the time period required for stabilization of measurements) and just before the start of DEX administration (TOI 1, nTHI 1); (ii) when the OAA/S score of 2 was reached after starting the initial loading (TOI 2, nTHI 2) and (iii) just before the start of treatment (TOI 3, nTHI 3), when the maintenance dose of DEX (0.2 μg/kg/h) was started. In the comparison of parameters between the MID and DEX groups, since the OAA/S score reached 1 (does not respond after mild prodding or shaking) in four patients in the DEX group, the values of the measured parameters at an OAA/S score of 2 (measurement point 2) were compared. All participants lay on the dental chair quietly with no stimulation (before starting dental treatments) after initiating data collection. Since treatment invasiveness differed depending on the dental treatment plan and the accuracy of BIS value could deteriorate by mixing with artifacts due to dental treatments and body movements, only measurements before treatment initiation were assessed.

Blood pressure was measured every 2.5 min. MAP, SpO_2_, etCO_2_ and BIS values obtained just before MID or DEX administration were used as the baseline values. For MAP, SpO_2_ and BIS values, the lowest value measured between the start of drug administration and achievement of an OAA/S score of 2 (measurement point 2) was recorded, and for etCO_2_, the highest value was recorded. Cognitive function was assessed using FAST and CDR after sedation, before the subjects went home. The assessments were conducted by a researcher (LM) other than the anesthesiologists.

### Statistical analysis

Statistical analysis was performed using SPSS version 16.0 software (SPSS Japan, Tokyo, Japan). Data are presented as medians (quartile). Comparisons of nTHI between the MID/DEX group and the DEX/MID group were analyzed using repeated measures analysis of variance for evaluating the validity of the drug sequences. The Wilcoxon test was used to compare parameters between the MID and DEX groups. For in-group comparisons, the Wilcoxon signed-rank test was used for MAP, SpO_2_, BIS and etCO_2_ values, as they were for before-and-after comparisons. TOI 1, 2, 3 and nTHI 1, 2, 3 comparisons were performed using the Friedman test, and as a post-hoc analysis, a Bonferroni-corrected Wilcoxon signed-rank test was performed. Multiple regression analysis was performed to examine independent associations between nTHI and other variables. Statistical significance was set at P < 0.05 (P < 0.017 after the Bonferroni correction).

The primary endpoint was the nTHI. A preliminary study was conducted to determine the required sample size as there are no previous studies comparing brain functions between MID and DEX administration in dementia patients. In the preliminary study, MID was administered intravenously (1.0 mg as the initial administration and 0.5–1.0 mg additional boluses, as required) in a total of four patients (≥ 60 years old) with dementia, or DEX was administered intravenously (3 μg/kg/h for 10 min) in a total of four elderly patients (≥ 60 years old) with dementia until an OAA/S score of 2 was reached, and the right nTHI (rnTHI) was measured at that time. The results showed that average values of rnTHI after MID or DEX administration were 0.89 ± 0.10 and 1.09 ± 0.20, respectively. The average standard deviation (0.151) was employed for calculating the required number of cases. According to calculations with the alpha error set at 5% and beta error at 20%, nine individuals per group were required to achieve a power of 80%. Assuming a drop-out rate of 20%, a final sample size of 11 patients in each group was required. However, the sample size in the current study was also decided according to a previous study on dementia that compared patients’ brain function parameters after administering two types of anesthetics, in which an associated factor, TOI, was compared between propofol and isoflurane administration. The results showed that TOI was 60 ± 7% in the propofol group and 67 ± 4% in the isoflurane group^17^. With the same alpha and beta error settings, 12 individuals per group were required to achieve a power of 80%. Estimating a drop-out rate of 20%, a final sample size of 14 patients in each group (total 14 patients) was required in this study. Based on these calculations, a sample size of 14 patients in each group (total 14 patients) was considered preferable and was therefore, employed in the present study. However, because two participants were found to have a history of cerebral infarction after enrollment and were subsequently excluded, a total of 12 patients were finally included in the present study.

## Results

### Patient background characteristics

A total of 12 patients (one man and 11 women) with a median age of 72 years (quartile 70.5 to 79.3 years), median height of 157.0 cm (153.8 to 157.5 cm), median weight of 54.9 kg (45.0 to 64.7 kg) and a median BMI of 25.1 kg/m^2^ (19.9 to 26.1) participated in the study (Table 1). There were 11 patients with Alzheimer-type dementia and one patient with Lewy body dementia. The FAST stages of the 11 Alzheimer-type dementia patients were as follows: three patients in stage 6e, one patient in stage 7a, four patients in stage 7b and three patients in stage 7c. The CDR for all was “severe”. The measured values were SpO_2_ 97 (96.3–98)%, etCO_2_ 36 (29–38) mmHg, MAP 92.5 (79.3–104.8) mmHg and BIS 93.5 (88–97) at baseline, when the participants underwent the first study procedures, indicating normal values of these parameters. Since a sedation level of OAA/S 2 was achieved in all patients who received 0.027 mg/kg of MID, no additional administration was required. However, while an OAA/S score of 2 (responds only after mild prodding or shaking) was achieved in eight patients after administration of 3 μg/kg/h DEX for 10 min, four patients eventually progressed to an OAA/S score of 1 (does not respond after mild prodding or shaking) after this initial DEX load. As an OAA/S score of 2 was achieved in all patients within 6–10 min of administration of 3 μg/kg/h DEX, each parameter was recorded when the OAA/S score reached 2 (second measurement). OAA/S scores of 2 were maintained in the MID group, and OAA/S scores of 2 or 1 were observed in patients in the DEX group when dental treatments were initiated (third measurement), which took 5–9 min after administering MID and 8–13 min after starting DEX administration.

**Table 1:** Characteristics of patients.

	Midazolam	Dexmedetomidine	P value	Mann–Whitney’s U value
Age (yo)	72 (70.5–79.3)			
Sex (male/female)	1/11			
Height (cm)	157.0 (153.8–157.5)			
Weight (kg)	54.9 (45.0–64.7)			
BMI	25.1 (19.9–26.1)			
Treatment time (min)	41.0 (30.5–51.3)	54.0 (46.8–78.8)	0.033	35.0
Sedation time (min)	66.0 (53.0–73.8)	98.5 (85.0–108.8)	< 0.001	5.5

### Comparison of patient background characteristics

Comparison of nTHI ([nTHI 2 on the right (rnTHI 2) and rnTHI 3, and nTHI 2 on the left (lnTHI 2) and lnTHI 3]) at each of the measurement points following MID and DEX administration between the MID/DEX group and the DEX/MID group indicated no differences (P > 0.297), and the drug sequence of MID and DEX was considered to not affect the results.

**Figure 3:**
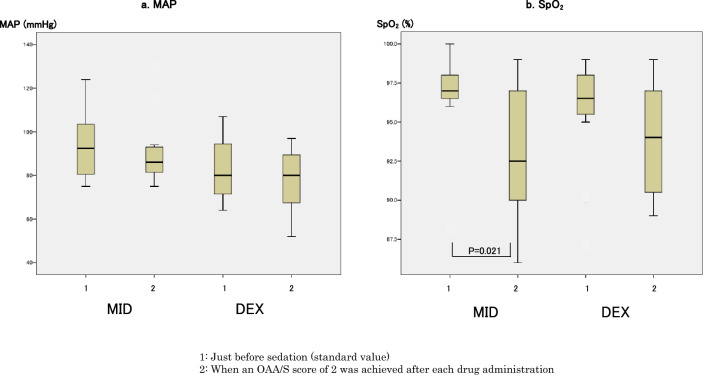
Changes in mean arterial pressure (MAP) and percutaneous arterial oxygen saturation (SpO_2_) values. (**a**) Mean arterial pressure (MAP) values were 92.5 (79.3–104.8) mmHg just before sedation and 86.0 (81.3–93.5) mmHg when a Modified Observer’s Assessment of Alertness/Sedation (OAA/S) score of 2 was achieved after midazolam administration in the midazolam group; and 80 (71.3–94.8) mmHg just before sedation and 80 (67.3–91.8) mmHg when an OAA/S score of 2 was achieved after dexmedetomidine administration in the dexmedetomidine group. There were no significant differences between the two measurement points in each group, and no significant differences at each measurement point between the two groups. (**b**) Percutaneous arterial oxygen saturation (SpO_2_) values were 97 (96.3–98)% just before sedation and 92.5 (89.5–97.5)% when an OAA/S score of 2 was achieved after midazolam administration (P = 0.021) in the midazolam group; and 96.5 (95.3–98)% just before sedation and 94 (90.3–97.5)% when an OAA/S score of 2 was achieved after dexmedetomidine administration in the dexmedetomidine group. There were no significant differences between the two measurement points in the dexmedetomidine group, and no significant differences at each measurement point between the two groups.

**Figure 4:**
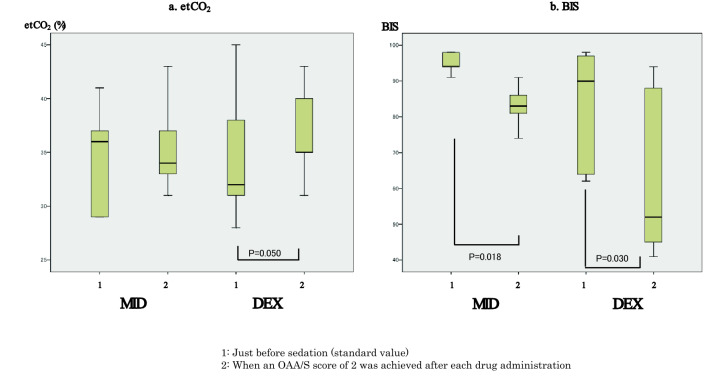
Changes in end-tidal CO_2_ (etCO_2_) and bispectral index (BIS) values. (**a**) End-tidal CO_2_ (etCO_2_) values were 36 (29–38) mmHg just before sedation and 34 (33–38) mmHg when a Modified Observer’s Assessment of Alertness/Sedation (OAA/S) score of 2 was achieved after midazolam administration in the midazolam group; and 33.5 (30.3–38.5) mmHg just before sedation and 35 (34.8–40.8) mmHg when an OAA/S score of 2 was achieved after dexmedetomidine administration in the dexmedetomidine group. There were no significant differences between the two measurement points in each group, and there were no significant differences at each measurement point between the two groups. (**b**) BIS values were 93.5 (88–97) just before sedation and 83 (78–89.8) when an OAA/S score of 2 was achieved after midazolam administration (P = 0.018) in the midazolam group; and 87.5 (62.5–96) just before sedation and 52 (44.5–82.5) when an OAA/S score of 2 was achieved after dexmedetomidine administration (P = 0.030) in the dexmedetomidine group. There were no significant differences at each measurement point between the two groups.

When the MID and DEX groups were compared, duration of treatment and sedation time in the DEX group were longer compared to those in the MID group (Table 1). There were no differences in MAP, SpO_2_, etCO_2_ and BIS values at each of the measurement points between the MID and DEX groups (Figs. 3, 4). In in-group comparisons of both drugs, the SpO_2_ value at an OAA/S score of 2 was significantly lower compared to baseline values in the MID group (Fig. 3). In both groups, BIS values at an OAA/S score of 2 were significantly lower compared to baseline values (Fig. 4), while there were no differences in MAP and etCO_2_ values at an OAA/S score of 2 (Figs. 3, 4).

## Results of NIRS measurements

### Changes in nTHI

#### Comparison between MID and DEX groups

**Figure 5:**
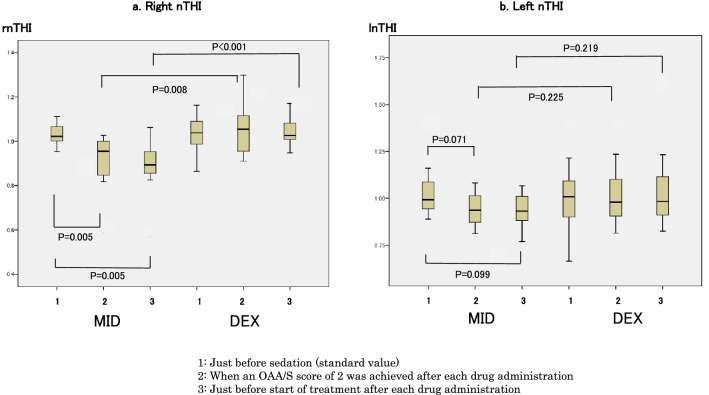
Changes in normalized tissue hemoglobin index (nTHI). (**a**) Right nTHI (rnTHI) values were 1.02 (1–1.08) just before sedation (baseline value), 0.95 (0.83–1) when a Modified Observer’s Assessment of Alertness/Sedation (OAA/S) score of 2 was achieved after midazolam administration, and 0.89 (0.86–0.96) just before start of treatment in the midazolam group; and 1.04 (0.96–1.12) just before sedation (baseline value), 1.05 (0.96–1.12) when an OAA/S score of 2 was achieved after dexmedetomidine administration, and 1.03 (1–1.08) just before start of treatment in the dexmedetomidine group. (**b**) Left nTHI (lnTHI) values were 0.99 (0.94–1.1) just before sedation (baseline value), 0.94 (0.86–1.02) when an OAA/S score of 2 was achieved after midazolam administration, and 0.93 (0.88–1.02) just before start of treatment in the midazolam group; and 1 (0.89–1.1) just before sedation (baseline value), 0.98 (0.90–1.12) when an OAA/S score of 2 was achieved after dexmedetomidine administration, and 0.98 (0.90–1.13) just before start of treatment in the dexmedetomidine group. In the midazolam group, since rnTHI values were significantly different between the three measurement points in patients with dementia (P = 0.001, χ^2^ = 13.83), post-hoc analysis was performed (P = 0.005 between rnTHI 1 and rnTHI 2; P = 0.005 between rnTHI 1 and rnTHI 3) (as indicated in the figure). Left nTHI values were not significantly different among the three measurement points in patients with dementia (P = 0.92, χ^2^ = 0.167). Comparison of right nTHI values at each time point (measurement points 2 and 3) between the midazolam and dexmedetomidine groups indicated significant differences between the two groups (P = 0.008 at measurement point 2 and P < 0.001 at measurement point 3 in the right nTHI group). The left nTHI values indicated no differences between the two drugs (P = 0.225 at measurement point 2 and P = 0.219 at measurement point 3).

Measurement of nTHI at the baseline point (rnTHI 1 and lnTHI 1) indicated baseline values of 1 in both groups. However, in rnTHI measurements, rnTHI 2 and rnTHI 3 in the MID group were each significantly lower than those in the DEX group (P = 0.008 and P < 0.001, respectively). However, in lnTHI measurements, lnTHI 2 and lnTHI 3 indicated no statistically significant difference between the two groups (Fig. 5).

#### Comparisons within each group

In the MID group, significant changes were observed in rnTHI values. In fact, rnTHI 2 and rnTHI 3 decreased significantly by − 5% and − 11%, respectively, compared to rnTHI 1 (baseline value = 1) (P = 0.001, χ^2^ = 13.83; post-hoc analyses were each P = 0.005 and P = 0.005, respectively). Left nTHI 2 and nTHI 3 decreased by − 6% compared to baseline; however, the difference was not significant (P = 0.092, χ^2^ = 0.167). All measurements on both sides in the DEX group exhibited the baseline value of 1 and no significant change was observed (Fig. 5).

### Changes in TOI

**Figure 6:**
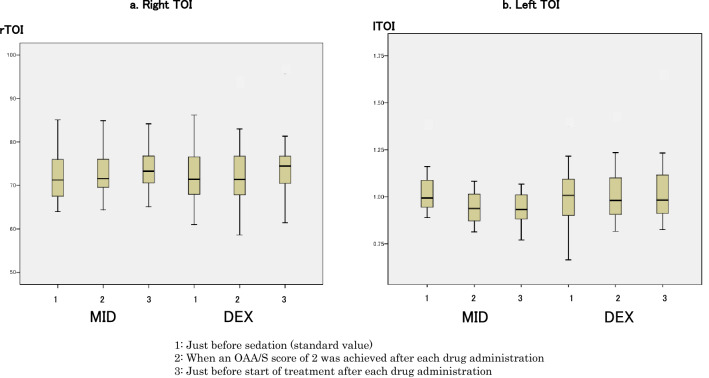
Changes in tissue oxygenation index (TOI). (**a**) Right TOI values were 71.3 (66.9–76.6)% just before sedation (baseline value), 71.6 (69.5–76.9)% when a Modified Observer’s Assessment of Alertness/Sedation (OAA/S) score of 2 was achieved after midazolam administration, and 73.3 (70.4–76.9)% just before start of treatment in the midazolam group; and 71.4 (67.5–78.6)% just before sedation (baseline value), 71.4 (67.4–79.2)% when an OAA/S score of 2 was achieved after dexmedetomidine administration, and 74.5 (70.2–77)% just before start of treatment in the dexmedetomidine group. (**b**) Left TOI values were 72.1 (70–76.7)% just before sedation (baseline value), 74.2 (70.6–76.4)%when an OAA/S score of 2 was achieved after midazolam administration, and 76.4 (73.6–79.8)% just before start of treatment in the midazolam group; and 75.3 (70.4–82.3)% just before sedation (baseline value), 74.7 (70.3–83.1)% when an OAA/S score of 2 was achieved after dexmedetomidine administration, and 77.8 (73.7–82.9)% just before start of treatment in the dexmedetomidine group. There were no significant differences in TOI values at each time point (measurement points 1, 2 and 3) between the midazolam and dexmedetomidine groups. Comparison of data at each time point (measurement points 1, 2 and 3) in each group indicated no differences.

There were no differences in TOI changes between the MID and DEX groups at each of the measurement points. There were also no significant changes within each group (Fig. 6).

### Independent associations between nTHI and the other variables

In multiple regression analysis in the MID group, the value of rnTHI 2, which decreased in the MID group at an OAA/S score of 2, was used as a dependent variable, whereas the independent variables included the measured values, such as the lowest values of SpO_2_ and MAP and the highest value of etCO_2_ at an OAA/S score of 2, since the values of these parameters can deteriorate during the course of sedation and affect CBF.

There were no factors associated with nTHI 2 in the MID group according to multiple regression analysis (P ≥ 0.082). This analysis was not performed in the DEX group since there were no changes in nTHI values in the DEX group.

### Changes in cognitive function

Cognitive function in both groups was assessed using FAST and CDR after sedation, before the subjects went home. None of the patients presented with reduced cognitive function.

## Discussion

In the present study, CBF decreased by up to 11% when elderly patients with severe dementia received intravenous sedation with 0.27 mg/kg of MID to achieve a sedation level of OAA/S 2. At the time of achievement of an OAA/S score of 2 (measurement point 2), CBF was significantly lower in the MID group (− 5%) than in the DEX group (± 0%). On the other hand, an OAA/S score of 2 or 1 was finally achieved in the DEX group during initial loading of 3 μg/kg/h of DEX for 10 min, although with maintenance of CBF. There was a comparable decrease in BIS values with both drugs.

The NIRS method used emits near-infrared light at three different wavelengths (775, 810, and 850 nm) into the brain tissue, and scattered reflected light from the three different near-infrared light sources irradiated into the tissue is measured at multiple locations of varying distances. Oxygenated hemoglobin, deoxygenated hemoglobin, total hemoglobin concentration, and the TOI were assessed by measuring the light attenuation rate. The nTHI as the ratio of the hemoglobin concentration at the measurement point to the total hemoglobin concentration at the start of measurement. The nTHI is known to sensitively reflect the CBF at the location on the forehead where the probes are placed^17,18^.

Tatsuno et al. found that MID sedation with a median dose of 0.035 mg/kg maintained CBF in elderly patients with no cognitive impairment, and MID sedation with a median dose of 0.027 mg/kg reduced CBF by approximately 10% in elderly patients with severe dementia^11^. Typically, as CBF is maintained at a steady level at a MAP range of 70 to 150 mmHg due to CBF autoregulation, absence of cognitive decline in response to MID administration in elderly patients due to maintenance of CBF despite MID administration is physiologically understandable^19^. On the other hand, many studies suggest that CBF reduction with MID is the result of suppression of cerebral metabolism, and that elderly patients with severe dementia might have weakened CBF autoregulatory mechanisms or already decreased cerebral metabolism, as was also observed in a previous study^11^.

Since DEX is an α2-agonist, it directly constricts vascular smooth muscles, thereby reducing CBF. In animal studies that assessed changes in CBF due to DEX, CBF decreased with 10 μg/kg of DEX administered to dogs anesthetized with inhalational anesthetics^20–22^. Additionally, DEX was shown to attenuate the cerebral vasodilation caused by volatile anesthetics^23^. Asano et al. reported a local reduction in CBF due to arterial constriction when DEX was locally administered to the pial arteries of rats anesthetized with isoflurane^24^. In a clinical study of healthy subjects, Zornow et al. observed a reduction in CBF and an increase in cerebrovascular resistance (CVR) when DEX was administered^25^. The dosage used in the study by Zornow et al. was consistent with the DEX dose used in the present study. Other researchers conducted studies using positron emission tomography or assessed middle cerebral artery blood flow velocity to measure CBF after initial loading with 1 μg/kg of DEX, and they reported a reduction in CBF, with a concomitant reduction in cerebral metabolic rate (CMR) respectively^26,27^. Hence, as described above, healthy human and animal studies have shown that DEX administration induces vasoconstriction and reduces CBF.

On the other hand, there are also studies that have reported on the cerebrovascular effects of DEX in the presence of brain damage. McPherson et al. reported that the vasodilatory response to hypoxia did not change after DEX administration in isoflurane-anesthetized dogs, and oxygen supply was maintained as a result of increased CBF due to the vasodilatory response to hypoxia^28^. Other researchers examined inhibition of the inflammatory response to DEX in mouse traumatic brain injury models, demonstrating cerebral protection through the inhibition of inflammatory responses by inflammatory cytokines, such as nuclear factor-kappa B, nucleotide-binding oligomerization domain-like receptor family pyrin domain-containing-3 and Toll-like receptor-4^29,30^. Matsumoto et al. measured plasma concentrations of norepinephrine and 3 methoxy-4-hydroxyphenethyleneglycol in rabbits in whom acute cerebral ischemia was induced, and reported decreased catecholamine concentrations with the administration of 250 μg/kg/h of DEX for 30 min^31^. In a clinical study, Farag et al. used 0.4 to 0.5 μg/kg/h of DEX sedation and 40 μg/kg/min of propofol sedation for surgeries, and measured cerebral tissue oxygenation with NIRS and CBF velocity, revealing that cerebral tissue oxygenation and CBF were comparable with both drugs^32^. Drummond et al. found that there was no reduction in brain partial pressure of oxygen when cerebrovascular surgery patients received an initial loading dose of 1 μg/kg of DEX for 10 min followed by a 15-min infusion of 0.5 to 0.7 μg/kg/h^33^. As shown above, DEX might have two effects. While DEX decreases CBF through vasoconstriction in the healthy brain, it, conversely, maintains CBF by suppressing catecholamine and inflammatory cytokine release in diseased brain tissues that have a low oxygen supply.

When elderly patients with severe dementia underwent intravenous sedation with DEX in the present study, nTHI measured with NIRS remained relatively stable compared to the levels before sedation, presenting more favorable results compared to MID, which resulted in 5% reduction (measurement point 2) or 11% reduction (measurement point 3) in nTHI. This suggests that CBF was maintained during sedation with DEX when an OAA/S score of 2 was achieved (measurement point 2). The same nTHI results were observed in the DEX group at measurement point 3 despite the fact that four of the 12 patients exhibited an OAA/S score of 1 (deeper sedation level) after the initial DEX load for 10 min.

In the absence of sudden brain damage, nTHI is thought to reflect blood flow in the part of the forehead where the probes are placed^15^. It is unclear whether a state of severe dementia is equivalent to the above-mentioned hypoxic condition or cerebral ischemic condition in the brain, and whether DEX suppresses catecholamines and inflammatory cytokines in the affected brain tissue in patients with dementia. However, the findings of the present study indicated that, in elderly patients with severe dementia, DEX sedation has a favorable effect on brain tissue compared to MID sedation.

Similar to the autoregulation of CBF by MAP, changes in PaO_2_ from 60 (equivalent to SpO_2_ 90%) to 300 mmHg have little influence on CBF^19^. CBF increases by 1 to 2 mL/100 g/min for each 1 mmHg increase in PaCO_2_ around normal PaCO_2_ values^19^. In this study, because SpO_2_ values decreased after MID administration, they might have affected CBF changes^19^. However, since multiple regression analysis proposed that rnTHI 2 did not correlate with these measured parameters, it suggests that CBF changes in this study were mainly affected by the choice of sedative (MID or DEX) used.

The results of the present study showed a comparable decrease in nTHI on both the left and right side when MID was administered to elderly patients with severe dementia. However, the analysis revealed no statistical significance in the decrease in lnTHI. Since one of the reasons might have been the small degree of decline in lnTHI 2 and lnTHI 3 compared to that of lnTHI 1, the results of the present study appear to be reasonable. However, a study on electroconvulsive therapy for schizophrenia reported a significant increase in lnTHI compared to rnTHI; thus, we cannot deny the possibility that there might be a bilateral difference in nTHI fluctuation depending on the inherent brain disorder and its location^18^.

BIS values, which are calculated using brain waves, showed a similar reduction in the MID and DEX groups, with no difference between the two groups. However, there was a large decrease in median BIS values in the DEX group (52) compared to the MID group (83) after the administration of both drugs. This was consistent with the report by Farag et al.^32^. A study of DEX sedation using brain waves (electroencephalography (EEG)) and electromagnetic tomography identified brain activity in the cuneus of the cerebral cortex and posterior cingulate cortex at characteristic frequency bands, indicating that DEX might exert a different cerebral response compared to other sedatives^34^. Therefore, since it is not clear whether a large decrease in BIS values accurately reflects the degree of sedation when using DEX, this should be investigated in future studies.

In our study, there were no changes in TOI in both the MID and DEX groups. Since TOI comprehensively assesses oxygen saturation in arteries and veins in the brain, it does not change rapidly, unlike arterial oxygen partial pressure and SpO_2_. In a previous report, anesthesia was induced with either a combination of midazolam and sufentanil or a combination of propofol and sufentanil, and the regional cerebral oxygen saturation (rSO_2_) measured by NIRS was recorded during induction. After pre-oxygenation, rSO_2_ significantly increased compared to baseline in each group, and did not show any additional increase after administration of either midazolam or propofol and sufentanil in each group. The report concluded that midazolam preserved cerebral oxygen supply–demand balance to a similar degree as propofol. It also reported that induction of general anesthesia resulted in further increase in rSO_2_ scores while administering a high inspired oxygen fraction^35^. In the present study, TOI probably did not change since 1 L/min of oxygen was administered to maintain oxygenation if SpO_2_ decreased to < 90%, and both midazolam and dexmedetomidine eventually preserved cerebral oxygen supply. This suggests that when severe dementia patients receive sedation with midazolam, supplemental oxygen should be given to maintain cerebral oxygenation despite the decrease in CBF.

Based on the fact that dementia grades (FAST and CDR) of the patients did not deteriorate after sedation with both drugs, we concluded that there is a decrease in BIS values, but no decline in cognitive function, with two exposures to sedatives among patients with severe dementia at FAST stages 6e to 7c and/or CDR indicating severe dementia. However, repeated sessions or a longer time course of sedation during dental treatment might affect cognitive function.

As the subjects of the present study were spontaneously breathing patients who did not undergo tracheal intubation, etCO_2_ measurements were made using a special nasal cannula for measuring CO_2_ and the relative values were compared. When the measurements were made prior to starting the dental treatment, the patient’s chin was lightly lifted to secure the airway, and the mouth was lightly closed manually to only measure CO_2_ exhaled from the nose. Hence, although the changes in relative values appear to be reasonable, the accuracy of the values might have been inferior to blood CO_2_ level measurements.

A limitation of the present study is that the methods of administration of the two sedatives were different. Measurement 2 data were obtained when an OAA/S score of 2 was indicated. However, some patients were sedated to an OAA/S score of 1 (deeper sedation level) when DEX was used (measurement 3). This could be related to different pharmacodynamic effects between the two drugs, inevitably leading to the different results. Based on these findings, the parameters were compared between both drugs at measurement point 2 (when an OAA/S score of 2 was achieved). Another limitation is that one of the factors of POCD is prolonged surgery, and, hence, the short duration of sedation (5–10 min) in the present study might have affected the cognitive deterioration. In this study, since treatment invasiveness differed depending on the dental treatment plan, only measurements before the start of treatment were assessed, which shortened the study time course. In the future, a longer time course study with the same treatment invasiveness in all the patients should be planned. The other limitations include frequent stimulation (calling and prodding) for evaluation of the OAA/S score in DEX group, which can impede the deepening of sedation levels. In this study, the stimulation for evaluation of the OAA/S score was performed within 10 s in each min, which was considered not to impede the deepening of sedation level. DEX is associated with fast recovery from sedation with physical stimulation if needed^5–9^, but clinical reports have shown that deep sedation with a BIS of < 60 can often be observed in patients with severe dementia who could not respond expressively to the stimulation^10^. Improved methods for evaluating sedation level need to be established and evaluated objectively in the future.

Other limitations are similar to those of our previous study^11^, namely that the probes for measuring brain waves (BIS) and NIRO^®^-200NX need to be placed on the forehead. Hence, while we could measure the brain waves and CBF at the forehead, the conditions in other parts of the brain were not known. Additionally, evaluation of the sedation level using the OAA/S score is highly subjective, which is a limitation. The BIS value does not necessarily accurately correlate with the sedation level^36,37^. Development of objective assessment methods will facilitate more accurate evaluation of the sedation level. Moreover, each category of dementia grade (FAST and CDR) has a certain range of symptoms. A subtle cognitive decline might result in the patient continuing to be classified in the same grade, due to the weak detection capability of FAST and CDR. Although this study satisfied the requirements of the statistical sample size calculation, the sample size was small, which is another limitation. Future large-scale studies should evaluate cerebral and cognitive function during and after sedation.

In conclusion, the present study demonstrated that CBF decreased up to 11% when elderly patients with severe dementia received intravenous sedation with MID to achieve a sedation level of OAA/S 2. When an OAA/S score of 2 was just achieved, CBF in the MID group (− 5%) was significantly lower than in the DEX group (± 0%). On the other hand, an OAA/S score of 2 or 1 was finally achieved in the DEX group during initial loading with 3 μg/kg/h of DEX for 10 min, although with maintenance of CBF. There was a comparable decrease in BIS values with both drugs. From the perspective of maintaining CBF, DEX might be a safer option for intravenous sedation in elderly patients with severe dementia.

## References

[1] Working Group on Guidelines Development for Intravenous Sedation in Dentistry, The Japanese dental Society of Anesthesiology (2018). Practice guidelines for intravenous conscious sedation in dentistry—Developed by The Japanese Dental Society of Anesthesiology for dentists practicing sedation in Japan. Anesth. Prog..

[2] White S (2019). Guidelines for the peri-operative care of people with dementia. Anaesthesia.

[3] Needham MJ, Webb CE, Bryden DC (2017). Postoperative cognitive dysfunction and dementia: What we need to know and do. Br. J. Anaesth..

[4] Vlisides PE, Avidan MS (2019). Recent advances in preventing and managing postoperative delirium. F1000Research..

[5] Pavone KJ, Cacchione P, Polomano RC, Winner LA, Compton P (2018). Evaluating the use of dexmedetomidine for the reduction of delirium: An integrative review. Heart Lung.

[6] Su X, Meng ZT, Wu XH, Cui F, Li HL, Wang DX, Zhu X, Zhu SN, Maze M, Ma D (2016). Dexmedetomidine for prevention of delirium in elderly patients after non-cardiac surgery: A randomised, double-blind, placebo-controlled trial. Lancet.

[7] Djaiani G, Silverton N, Fedorko L, Carroll J, Styra R, Rao V, Katznelson R (2016). Dexmedetomidine versus propofol sedation reduces delirium after cardiac surgery. Anesthesiology.

[8] Yang W, Kong LS, Zhu XX, Wang RX, Liu Y, Chen LR (2018). Effect of dexmedetomidine on postoperative cognitive dysfunction and inflammation in patients after general anesthesia. Medicine.

[9] Bekker A, Sturaitis MK (2005). Dexmedetomidine for neurological surgery. Neurosurgery.

[10] Nishizaki H, Morimoto Y, Hayashi M, Iida T (2021). Analysis of intravenous sedation for dental treatment in elderly patients with severe dementia—A retrospective cohort study of a Japanese population. J. Dent. Sci..

[11] Tatsuno Y, Morimoto Y, Hayashi M, Iida T (2021). Comparison of intravenous sedation using midazolam during dental treatment in elderly patients with/without dementia: A prospective, controlled clinical trial. Sci. Rep..

[12] Tsoi KF, Chan JY, Hirai HW, Wong SY, Kwok TC (2015). Cognitive tests to detect dementia: A systematic review and meta-analysis. JAMA Intern. Med..

[13] Crevoisier C, Ziegler WH, Eckert M, Heizmann P (1983). Relationship between plasma concentration and effect of midazolam after oral and intravenous administration. Br. J. Clin. Pharmacol..

[14] Maruishi Pharmaceutical Co (2020). The Medical Package Insert of Precedex®.

[15] Yoshitani K, Kawaguchi M, Ishida K, Maekawa K, Miyawaki H, Tanaka S, Uchino H, Kakinohana M, Koide Y, Yokota M, Okamoto H, Nomura M (2019). Guidelines for the use of cerebral oximetry by nearinfrared spectroscopy in cardiovascular anesthesia: A report by the cerebrospinal Division of the Academic Committee of the Japanese Society of Cardiovascular Anesthesiologists (JSCVA). J. Anesthesia.

[16] Miller RD (2015). Intravenous Anesthetics in Miller’s Anesthesia.

[17] Yoshitani K, Kawaguchi M, Tatsumi K, Sasaoka N, Kurumatani N, Furuya H (2004). Intravenous administration of flurbiprofen does not affect cerebral blood flow velocity and cerebral oxygenation under isoflurane and propofol anesthesia. Anesth. Analg..

[18] Fujita Y, Takebayashi M, Hisaoka K, Tsuchioka M, Morinobu S, Yamawaki S (2011). Asymmetric alternation of the hemodynamic response at the prefrontal cortex in patients with shizophrenia during electroconvulsive therapy: A near-infrared spectroscopy study. Brain Res..

[19] Patel PM, Drummond JC, Lemkuil BP, Miller RD (2015). Cerebral physiology and the effects of anesthetic drugs. Mialler’s Anesthesia.

[20] Karlsson BR, Forsman M, Roald OK, Heier MS, Steen PA (1990). Effect of dexmedetomidine, a selective and potent α2-Agonist on cerebral blood flow and oxygen consumption during halothane anesthesia in dogs. Anesth. Analg..

[21] Zornow MH, Fleischer JE, Scheller MS, Nakakimura K, Drummond JC (1990). Dexmedetomidine, an α2-adrenergic agonist, decreases cerebral blood flow in the isoflurane-anesthetized dog. Anesth. Analg..

[22] Fale A, Kirsch JR, McPherson RW (1994). α2-adrenergic agonist effects on normocapnic and hypercapnic cerebral blood flow in the dog are anesthetic dependent. Anesth. Analg..

[23] Ohata H, Iida H, Dohi S, Watanabe Y (1999). Intravenous dexmedetomidine inhibits cerebrovascular dilation induced by isoflurane and sevoflurane in dogs. Anesth. Analg..

[24] Asano Y, Koehler RC, Kawaguchi T, Mcpherson RW (1997). Pial arteriolar constriction to α2-adrenergic agonist dexmedetomidine in the rat. Am. J. Physiol..

[25] Zornow MH, Maze M, Dyck JB, Shafer SL (1993). Dexmedetomidine decreases cerebral blood flow velocity in humans. J. Cereb. Blood Flow Metab..

[26] Prielipp RC, Wall MH, Tobin JR, Groban L, Cannon MA, Fahey FH, Gage HD, Stump DA, James RL, Bennett J, Butterworth J (2002). Dexmedetomidine-induced sedation in volunteers decreases regional and global cerebral blood flow. Anesth. Analg..

[27] Drummond JC, Dao AV, Roth DM, Cheng CR, Atwater BI, Minokadeh A, Pasco LC, Patel PM (2008). Effect of dexmedetomidine on cerebral blood flow velocity, cerebral metabolic rate, and carbon dioxide response in normal humans. Anesthesiology.

[28] McPherson RW, Koehler RC, Traystman RJ (1994). Hypoxia, α2-adrenergic, and nitric oxide-dependent interactions on canine cerebral blood flow. Am. J. Physiol..

[29] Wang D, Xu X, Wu YG, Lyu L, Zhou ZW, Zhang JN (2018). Dexmedetomidine attenuates traumatic brain injury—Action pathway and mechanisms. Neural Regen. Res..

[30] Yamanaka D, Kawano T, Nishigaki A, Aoyama B, Tateiwa H, Locatelli MS, Locatelli FM, Yokoyama M (2017). Preventive effects of dexmedetomidine on the development of cognitive dysfunction following systemic inflammation in aged rats. J. Anesth..

[31] Matsumoto M, Zornow MH, Rabm BC, Maze M (1993). The α2-adrenergic agonist, dexmedetomidine, selectively attenuates ischemia-induced increases in striatal norepinephrine concentrations. Brain Res..

[32] Farag E, Kot M, Podolyak A, Argalious M, Deogaonkar M, Mascha EJ, Xu Z, Katzan I, Ebrahim Z (2017). The relative effects of dexmedetomidine and propofol on cerebral blood flow and regional brain oxygenation—A randomized noninferiority trial. Eur. J. Anaesthesiol..

[33] Drummond JC, Sturaitis MK (2010). Brain tissue oxygenation during dexmedetomidine administration in surgical patients with neurovascular injuries. J. Neurosurg. Anesthesiol..

[34] Kim WH, Cho D, Lee B, Song JJ, Shin TJ (2017). Changes in brain activation during sedation induced by dexmedetomidine. J. Int. Med. Res..

[35] Kim DH, Kwak YL, Nam SH, Kim MS, Kim EM, Shim JK (2009). Assessment of cerebral oxygen supply-demand balance by near-infrared spectroscopy during induction of anesthesia in patients undergoing coronary artery bypass graft surgery: Comparison of midazolam with propofol. Korean J. Anesthesiol..

[36] Shetty RM (2018). BIS monitoring versus clinical assessment for sedation in mechanically ventilated adults in the intensive care unit and its impact on clinical outcomes and resource utilization. Cochrane Database Syst. Rev..

[37] Lim TW (2019). Efficacy of the bispectral index and Observer’s Assessment of Alertness/Sedation Scale in monitoring sedation during spinal anesthesia: A randomized clinical trial. J. Int. Med. Res..

