# Author Correction: Comparison of intravenous sedation using midazolam versus dexmedetomidine in elderly patients with dementia: a randomized cross-over trial

**DOI:** 10.1038/s41598-022-12641-3

**Published:** 2022-05-25

**Authors:** Yoshinari Morimoto, Megumi Hayashi, Yuki Yao, Hitomi Nishizaki, Hidechika Ishii, Lou Mikuzuki, Kouji Hara

**Affiliations:** Division of Geriatric Dentistry, Department of Critical Care Dentistry, Kanagawa Dental University, 82, Inaoka-cho, Yokosuka, Kanagawa 238-8580 Japan

Correction to: *Scientific Reports* 10.1038/s41598-022-10167-2, published online 15 April 2022

The original version of this Article contained an error in Figure 6 where the error bars were omitted in panel (a). The original Figure 6 and accompanying legend appear below.

**Figure 6:**
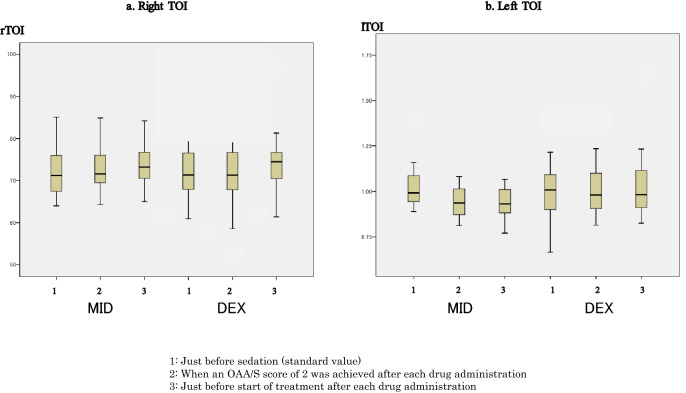
Changes in tissue oxygenation index (TOI). (**a**) Right TOI values were 71.3 (66.9–76.6)% just before sedation (baseline value), 71.6 (69.5–76.9)% when a Modified Observer’s Assessment of Alertness/Sedation (OAA/S) score of 2 was achieved after midazolam administration, and 73.3 (70.4–76.9)% just before start of treatment in the midazolam group; and 71.4 (67.5–78.6)% just before sedation (baseline value), 71.4 (67.4–79.2)% when an OAA/S score of 2 was achieved after dexmedetomidine administration, and 74.5 (70.2–77)% just before start of treatment in the dexmedetomidine group. (**b**) Left TOI values were 72.1 (70–76.7)% just before sedation (baseline value), 74.2 (70.6–76.4)%when an OAA/S score of 2 was achieved after midazolam administration, and 76.4 (73.6–79.8)% just before start of treatment in the midazolam group; and 75.3 (70.4–82.3)% just before sedation (baseline value), 74.7 (70.3–83.1)% when an OAA/S score of 2 was achieved after dexmedetomidine administration, and 77.8 (73.7–82.9)% just before start of treatment in the dexmedetomidine group. There were no significant differences in TOI values at each time point (measurement points 1, 2 and 3) between the midazolam and dexmedetomidine groups. Comparison of data at each time point (measurement points 1, 2 and 3) in each group indicated no differences.

The original Article has been corrected.

